# A16 ALTERATIONS TO BOTH INTESTINAL BARRIER INTEGRITY AND THE MICROBIOTA CONSPIRE DURING AGING TO PROMOTE DISEASE PROGRESSION IN A MOUSE MODEL OF MULTIPLE SCLEROSIS

**DOI:** 10.1093/jcag/gwad061.016

**Published:** 2024-02-14

**Authors:** L S Hohman, N M Fettig, A Pu, F Schroeder, J Gommerman, L Osborne

**Affiliations:** The University of British Columbia, Vancouver, BC, Canada; The University of British Columbia, Vancouver, BC, Canada; University of Toronto, Toronto, ON, Canada; Cornell University, Ithaca, NY; University of Toronto, Toronto, ON, Canada; The University of British Columbia, Vancouver, BC, Canada

## Abstract

**Background:**

Multiple sclerosis (MS) is a chronic, inflammatory condition impacted by both genetic and environmental factors. Aged individuals have an increased risk of progressive multiple sclerosis (pMS), as well as altered microbiota community composition and elevated levels of circulating gut microbiota-derived metabolites due to compromised gut barrier function. Fecal microbiota transplantation (FMT) studies confirm that an MS-associated microbiome can potentiate disease onset and enhance neuroinflammation in experimental autoimmune encephalomyelitis (EAE), a murine model of MS.

**Aims:**

1) Assess whether an aged microbiota alone is sufficient to promote age-associated progressive-like disease in our mouse model of EAE

2) Determine whether altered intestinal barrier integrity results in central nervous system-specific immune changes and/or disease course during EAE

**Methods:**

To assess the impact of an aged microbiota on EAE outcomes we performed age-disparate human-derived FMT from young vs aged healthy co-habiting, first degree relatives into recipient mice. Then, to further interrogate the role of the gut-brain axis in the association of age and pMS we isolated the compromised intestinal barrier seen in aged individuals as a variable via two methods – Muc2^-/-^ mice, which exhibit a defective mucus barrier, and DSS treated mice.

**Results:**

Following adoptive transfer of young EAE-primed T cells, ‘young’ FMT recipients exhibited disease resolution while an ‘aged’ FMT lead to severe, non-remitting EAE relative to both controls and ‘young’ FMT recipients. This difference in outcomes was not seen in all donor pairs, suggesting that worsened EAE is not a universal feature of the aged microbiota. We collected cecal material, blood, and central nervous system (CNS) tissue from an age-differential FMT mouse pair and performed metabolomic analysis. We found robust differences in the metabolomic profile of young vs aged FMT recipients before and after EAE induction. Further, in both models of altered barrier integrity, we observed altered myeloid cell trafficking to and immune activation in the CNS, suggesting that the intestinal barrier itself plays a critical role in immune activation during EAE.

**Conclusions:**

These findings suggest specific age-related microbiota-derived metabolites, which have increased access to the host system due to a leaky intestinal barrier, may be associated with increased risk of progressive disease and may represent promising therapeutic targets for MS.

**Funding Agencies:**

CIHRMS Canada

